# Impact of different restorative treatments for deep caries lesion in primary teeth (CEPECO 1) – study protocol for a noninferiority randomized clinical trial

**DOI:** 10.1186/s12903-018-0703-3

**Published:** 2019-01-08

**Authors:** Gabriela Seabra Quennehen da Silva, Daniela Prócida Raggio, Gabriela Fernanda Ribeiro Machado, Anna Carolina Volpi Mello-Moura, Thais Gimenez, Isabela Floriano, Tamara Kerber Tedesco

**Affiliations:** 1 Graduation Program, School of Dentistry, Ibirapuera University, Av. Interlagos 1329, São Paulo, SP 04661-100 Brazil; 2 Department of Orthodontic and Pediatric Dentistry, School of Dentistry, University of São Paulo, Av. Prof. Lineu Prestes, 2221, São Paulo, SP 05508-000 Brazil; 3 School of Dentistry, UNINOVAFAPI University Centre, Rua Vitorino Orthiges Fernandes, 6123, Teresina, PI 64073-505 Brazil

**Keywords:** Pediatric dentistry, Dental caries, Primary teeth

## Abstract

**Background:**

Due to the lack of evidence to determine the best treatment for deep cavitated caries lesions in primary molars, the search for an effective restorative technique, which results in a minimal discomfort to patients, and reduce the time needed for the treatment, becomes relevant. The objective of this randomized clinical trial was to evaluate if high-viscosity glass ionomer cement (HVGIC) restorations is noninferior to restoration with calcium hydroxide cement associated with HVGIC for treatment of deep lesions in primary molars, as well as the impact of the treatments on cost and discomfort of the patient.

**Methods:**

A non-inferiority randomized clinical trial with two parallels arms (1:1) will be conducted. Children with 4 to 8 years will be selected at Clinic of Pediatric Dentistry at Ibirapuera University. 108 teeth will be randomized into two groups: (1) Calcium hydroxide cement associated with HVGIC and (2) HVGIC restoration. Primary outcome will be considered the pulp vitality and to be evaluated after 6, 12, 18 and 24 months by two calibrated examiners. Survival of restorations will also be evaluated in the equal intervals. The duration of dentals treatment and the cost of all materials used will be considered for estimating of cost-efficacy of each treatment. Individual discomfort will be measured after each dental procedure using the Wong-Baker’s Facial Scale. For the primary outcome, Kaplan-Meier survival and the long-rank test will be used to comparison between the groups. Cox regression will be performed to assess the influence of variables on the outcome. For all analyzes, the significance level is set at 5%.

**Discussion:**

Based on the philosophy of ART, our hypothesis is that the HVGIC restoration is a possible approach to restore the deep caries lesion with pulp vitality without the use of rubber dam and anesthesia.

**Trial registration:**

Clinicaltrials.gov registration NCT02903979. Registered on June 9th 2016.

**Electronic supplementary material:**

The online version of this article (10.1186/s12903-018-0703-3) contains supplementary material, which is available to authorized users.

## Background

In Pediatric Dentistry, a number of factors have contributed to the marked decline in dental caries rates [1, 2]. However, this is a disease that still deserves attention, given its involvement in all age groups and, mainly, its negative impact on children’s quality of life [3, 4].

With the better biological understanding of the disease, as well as the importance of the etiological and modifiers factors, new concepts were developed for treating of these lesions, especially those already cavitated, in order to use less invasive restorative techniques and preventive approaches [5]. These changes in the paradigms allow, therefore, the accomplishment of more conservative cavity preparations, with significant preservation of enamel and dentin, since it is possible only the removal of the irreversibly affected tissues by the caries lesion [6, 7].

The treatment of deep caries lesions closes to the pulp considered healthy, on the other hand, results in a challenge for the dentists [8], especially for a gap in well-designed studies that determine the best treatment for these lesions [9].

Considering the requirements of Minimal Intervention dentistry, indirect pulp capping has been described as an effective option for the treatment of these lesions [8]. Based on the technique of selective dentin caries removal, indirect pulp capping is performed in a single dental session and aims to use a biocompatible material to protect the dentin-pulp complex, such as calcium hydroxide cement [8], which would have as benefits the reduction of the number of remaining bacteria as well as a possible dentinal response leading to the formation of a reparative dentin [10]. Recent studies still have suggested the use of inert materials for this protection because they would also have the capacity to arrest the caries process [11] or, even, the direct restoration of the cavity with adhesive systems associated with resin composite [12] or resin-modified glass ionomer cement [13].

Nevertheless, the high viscosity glass ionomer cement (HVGIC), which has been the material of choice for medium and low deep cavities in Atraumatic Restorative Treatment (ART), has not been considered in the studies that focusing in treatment of deep caries lesion [14]. Using this material in pediatric dentistry seems to be an alternative to decrease the time required for the clinical care, due to the facility to perform the restorations with HVGIC. Considering the philosophy of ART, it will be possible to restore the deep caries lesion with pulp vitality without the use of rubber dam and anesthesia. However, to date, there are no well-designed clinical trials evaluating cost-efficacy as well as other important patient-centered outcomes of the treatment of deep caries lesions with HVGIC.

Thus, this study aims as primary outcome to compare the pulp vitality of two types of treatment for deep caries lesions in primary molars (HVGIC restoration and restoration with calcium hydroxide cement associated with HVGIC) by a non-inferiority randomized clinical trial with two parallel arms. The secondary outcomes will compare the survival of restorations, caries progression, cost-efficacy and discomfort between the two treatment options. Our hypothesis is that the dental pulp vitality of teeth restored with HVGIC do not differ from teeth restored with a pulp capping material.

## Methods/design

### Trial design and ethical considerations

A non-inferiority randomized, controlled, double blind (participant and examiner) clinical trial with two parallels arms (1:1) will be performed. The present protocol follows the guidelines of the Standard Protocol Items: Recommendations for Interventional Trials (SPIRIT) as detailed in online Additional file 1.

It was approved by the local ethics committee from the Faculty of Dentistry of the University Ibirapuera (registration no. 1.670.059) and was recorded in the database for registration of clinical studies (Clinicaltrials.gov registration NCT02903979).

### Sample size calculation and selection

Participants will be selected with ages ranging from 4 to 8 years searching for dental treatment, coming from the Clinic of Pediatric Dentistry of the Ibirapuera University, Sao Paulo, Brazil. The screening will be carried out under natural light with the aid of a wooden spatula. Children with potential for inclusion in the research will be referred for clinical examination. Children with at least one primary molar with deep cavitated caries lesion on the occlusal or occlusoproximal surfaces will be included.

Special needs patients using orthodontic appliance and / or systemic diseases that may influence the oral cavity, will be excluded. In addition, teeth with pulp exposure, spontaneous pain, mobility, abscess or fistula near the tooth, teeth with restorations, sealants or defects of enamel formation will be excluded.

Initial periapical radiography will also be performed to confirm the depth of the lesions as well as to exclude a possible pulpal involvement – presence of radiographically visible furca/periapical lesion - linked to clinical evaluation by PUFA index [15] - presence of ulceration, fistula and abscess, reported pain and pathological mobility. It will be considered as deep caries lesion those that will be located in the inner third of dentin.

To perform the sample size calculation, the expected success rates of pulp vitality using a calcium hydroxide cement as pulp capping material was considered 94% in 12–29 months [16]. It was considered that a clinically significant difference was 15% in the success rate between the groups. Therefore, considering a significance level of 0.05 and a power of 0.80, using a one-tailed test for non-inferiority studies, with a 20% increase due to a possible sample loss and 40% by cluster of more than one tooth per children, we reached the final rounded number of 54 teeth per group, resulting in 108 teeth in total [17].

**Fig. 1:**
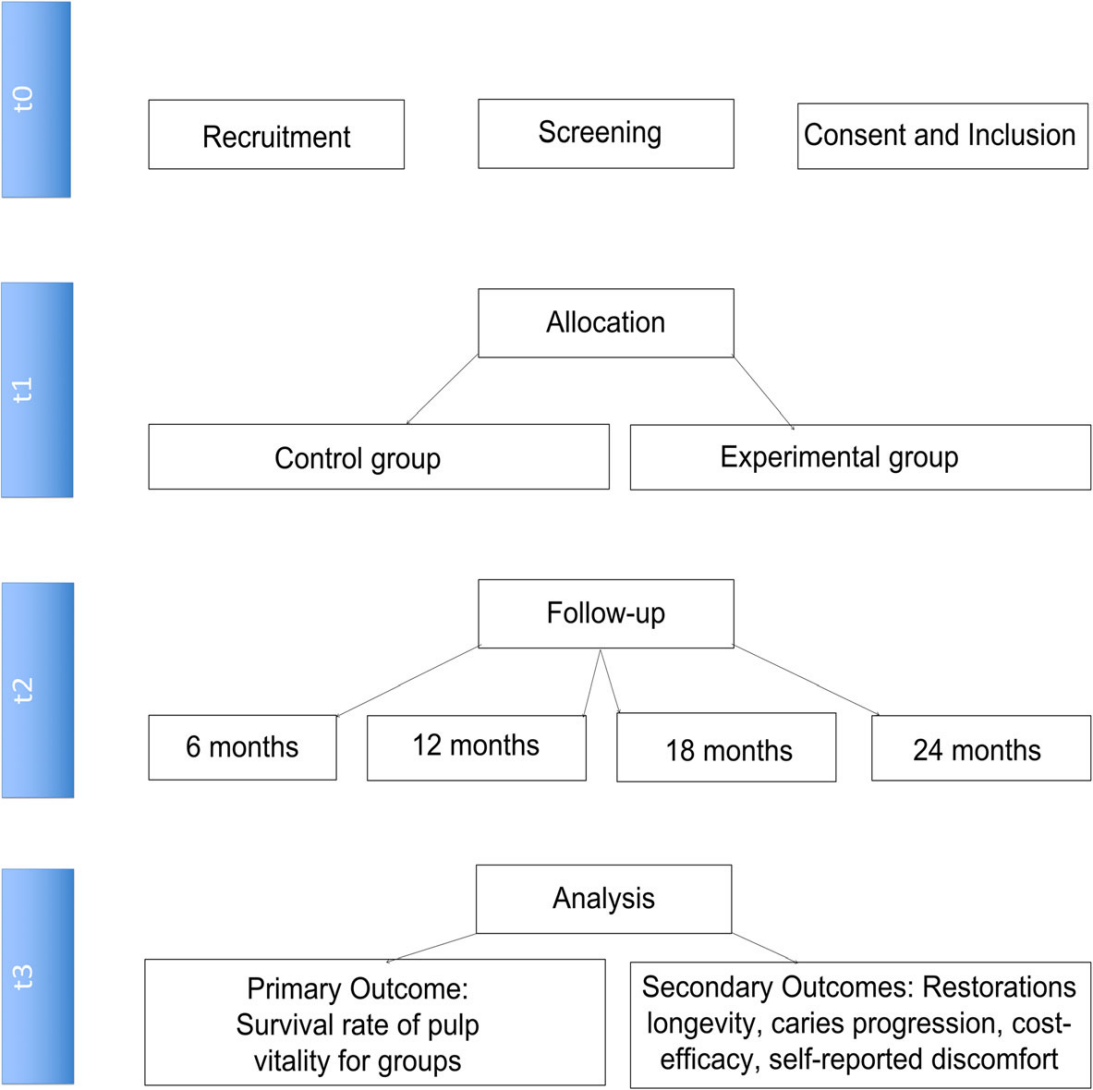
Flow diagram of clinical trial’s phases

Recruitment are taking place from November 2016 to April 2018. After allocation and treatment in one of the groups, with mean of 1 month per participant, these will be followed up for 24 months. Figure 1 displays the flow diagram of clinical trial’s phases.

### Operator’s training

Prior to sample selection, operators will be trained to perform both techniques (restoration with HVGIC and restoration with hydroxide calcium cement associated with HVGIC). The training will be performed with theoretical classes and laboratory activities during 3 h each.

The operators will be specialists in Pediatric dentistry. It will not be possible the blinding of operators because of the evident differences between the techniques.

### Random allocation

The included teeth will be allocated in two parallels arms: Experimental group – HVGIC restoration, e Control group - restoration with calcium hydroxide cement associated with high viscosity glass ionomer cement (HVGIC). Teeth will be randomly assigned into one of groups considering the strata type of cavity – occlusal or occlusoproximal surfaces, according with the sequence obtained by an external researcher with a statistical software (MedCalc version 15.8, Ostend, Belgium). The randomization procedure will be performed per blocks of four. Table 1 displays the sample distribution in according experimental groups considering the strata.

**Table 1:** Sample distribution in according experimental groups considering the strata

Groups Type of cavity	Experimental group	Control groups	Total
Occlusal	27	27	54
Occlusoproximal	27	27	54

### Allocation concealment mechanism

The generated sequence will be distributed in numbered sequentially opaque sealed envelopes, which should be opened by the dental assistant immediately before of the restorative procedure, after selective caries removal.

### Treatment procedures

#### HVGIC restoration

In experimental group, after prophylaxis and relative isolation, selective dentin caries removal will be conducted, removing infected dentin from the pulp wall and with total removal of the surrounding walls, using curettes compatible with cavity size. Afterwards, the preconditioning of the surface with polyacrylic acid for 10 s will be performed, followed by washing and drying of the cavity. HIVGIC (Fuji IX; GC Corporation, Tokyo, JP) will be mixed according to manufacturer’s instructions, inserted into the cavity with the aid of an insertion spatula and adapted by the finger press technique. In occlusoproximal cavities, metal matrix will be used to ensure the contact area between the restored and adjacent teeth. The occlusion will be then checked with carbon paper and, if necessary, occlusal adjustment will be performed. Superficial protection of the restoration with petroleum jelly will be conducted.

#### Restoration with hydroxide calcium cement associated with HVGIC

In control group, after prophylaxis and relative isolation, selective dentin caries removal will be conducted in according with experimental group, and then a pulp capping material (Hydro C; Sirona, Pennsylvania, USA) will be applied as liner on pulp wall. After, the restoration with HVGIC will be performed as previously mentioned for experimental group.

**Fig. 2:**
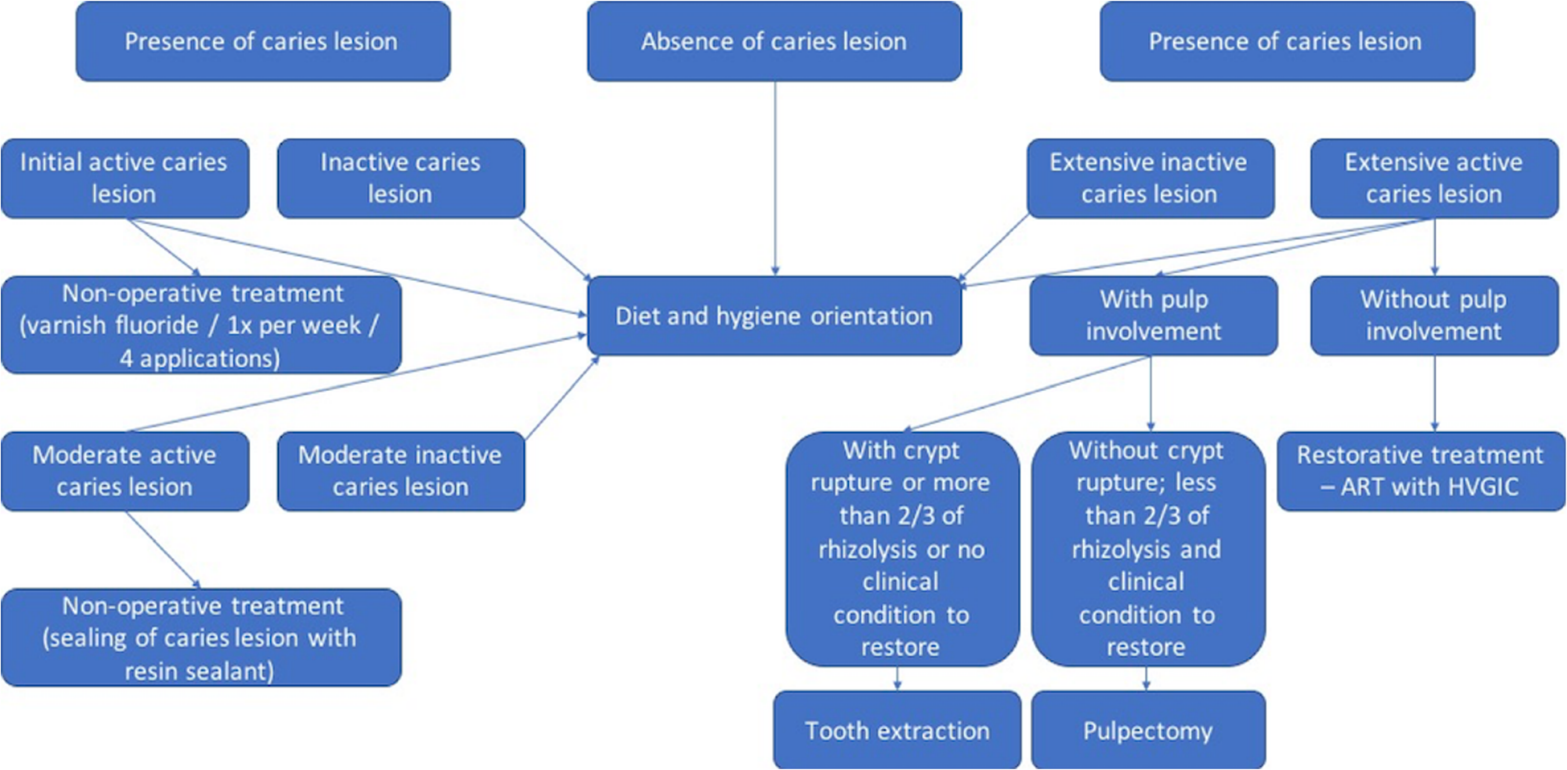
Organization chart of decision-making process of teeth not included in the trial

The other teeth identified with caries lesions that will be not included in the study will be treated according to the diagnosis by participants of CEPECO collaborative group (Fig. 2).

Furthermore, all participants and their respective legal guardians will receive hygiene and diet instructions. The risks for the participants of this study are minimal and related to the conventional treatment for deep caries lesion. There is no data monitoring committee.

### Data collection and outcomes

Confidentiality of participants will be ensured by an identifier number. Data will be stored in a password-protected electronic database by one of investigators, which only will be available to the researchers. Another investigator will go then double-checking of entered data.

The primary outcome will be the success rates of pulp vitality for both groups after follow-up for 2 years. Secondary outcomes will include survival of restorations, caries progression, self-reported discomfort and cost-efficacy of both types of restorative treatment.

Two blinded examiners will be trained to outcomes assessment. The training will be performed in two phases:

1. Theories classes with images during 3 h.
2. Clinical phase with children with similar conditions to be considered in trial, but not included, during 20 h.

#### Primary outcome

##### Pulp vitality

Pulp vitality will be evaluated after 6, 12, 18 and 24 months through clinical examination using PUFA index [15] linked to radiographic analysis. It will be considered success when minor failures will be observed (failures which could be resolved by replacing or repair of failed restoration). Failure of treatment will be pondered in the presence of major failures as visible pulp involvement, ulceration, fistula and abscess. Reported pain and pathological mobility will also be contemplated. Moreover, we will be considered major failure when the teeth will present radiographically visible furca/periapical lesion.

#### Secondary outcome

##### Survival of restorations

Survival of restorations will be evaluated after 6, 12, 18 and 24 months through of new clinical examination using the criteria by Frencken et al. [14] to occlusal cavities and that proposed by Roeleveld et al. [18] for occlusal-proximal restorations. In occlusal restorations those that receive 0, 1 or 7 score will be considered as success, whilst for occlusoproximal cavities it will be considered as success only those that show 00 or 10 scores. In failures cases, it will be registered the number of surfaces involved in the caries progression and the repair of restoration will be performed.

##### Caries progression

For the evaluation of the caries progression, the bitewing radiographic examination will be used. Radiographic shots will follow the protocol: it will be used a children’s E-speed film (E-speed, 22x35mm, Eastmam Kodak, Rochester, USA), with 0.4 s of exposure, apparatus with Spectro 70X. All radiographs will be made with positioners (Jon Han-Shin PF 682, Jon Ind., Sao Paulo, BRA), apron and lead collar. The films will be processed in boxes of manual processing by the time / temperature method (temperature around 27 °C, developer solution for 2 min, fixer solution for 10 min, washing in water for 20 min). Three radiographs per patient will be performed (1. At the initial exam, 2. After the restorative procedure, 3. Follow-up after 24 months). Initially, two examiners previously trained and calibrated by a reference examiner will evaluate the radiographs independently. The follow ups radiograph will then be compared with post-operative radiograph in order to assess a possible caries lesion progression:

A) No progression: when there is no increase in the radiolucent area of the lesion.
B) Present progression: when there is an increase in the radiolucent area of the lesion.

##### Self-reported discomfort

The child will also be questioned about the discomfort in relation to the treatment performed. For this purpose, the Wong-Baker face scale [19] will be used. This will be showed by the dental assistant, without the presence of the operator, immediately after treatment, and the child will point to the image which represents your level of discomfort after the following question: what did you feel during the treatment?.

##### Cost-efficacy

The number of expected and unexpected visits for each patient (indirect costs), the procedure performed at each session and their duration will be considered in the analysis. To calculate the direct costs, it will be computed the costs of all material used. These values will be based on the market value obtained by an average cost by three different stores of dental materials and this data will be updated during the study [20]. The duration of the treatments (time of treatment) and the cost of the materials used will be considered for the estimation of the cost-efficacy of the treatments by a ratio - cost/efficacy, being efficacy considered the pulp vitality.

##### Data analysis

The efficacy of each treatment will be assessed by five main outcomes:

1. Success rate of pulp vitality (Primary outcome) and
2. survival of restorations (secondary outcome): Kaplan-Meier survival and the Long-rank test will be used to compare the success rates between the experimental and control groups. Cox regression model with a shared frailty will be performed in order to allow the evaluation of the influence of the variables in the results.
3. Progression of deep caries lesions (secondary outcome): Qui-square test will be used to compare this outcome between the groups.
4. Cost-efficacy (secondary outcome): Incremental cost-efficacy ratio will be calculated considering the ratio between the total cost of each treatment and the success rate after 2 years.
5. Self-reported discomfort (secondary outcome): Poisson regression will be used to compare both groups and to asses of the influence of other variables on this outcome.

For all analyzes, the significance value will be adjusted to 5%.

### Patient and public involvement

Children treated by ART philosophy have pointed for less anxiety, pain and discomfort with minimally invasive techniques, as previously studies showed [21]. Thus, this preference reported by them helped to guide this trial. The research question emerged from the lack of evidence of the use of ART philosophy for deep cavitated caries lesions in primary molars, both for efficacy, as well as the discomfort caused to patients.

Because it is a clinical study methodology, the patients are still being involved in the recruitment, conduction and evaluation of the study. However, there is no involvement of patients or public in trial design.

The authors intend to disseminate the results of the present study not only through scientific publications, but also with public and private clinicians.

### Ethics and dissemination plain

This clinical trial was approved by the local ethical board committee from the Faculty of Dentistry of the Ibirapuera University (registration no. 1.670.059). Participants will be included after their legal guardians have signed an informed consent form containing detailed information about the research and the children nod their participation. The data from participants’ individual information will be grouped in order to prevent individual participant identification and then will be published in a peer-reviewed journals and presented at conferences.

## Discussion

Studies focusing in smart and comfortable techniques to children’s treatment should be conducted in order to guide the dentists to use of friendly-patient approaches. Thus, this is the first clinical trial that will evaluate as primary outcome the success rate considering the pulp vitality between restoration with calcium hydroxide cement associated with HVGIC and HVGIC restoration for treatment of deep caries lesions in primary molars in according the philosophy of ART.

The evaluation of this outcome will take into account clinical signs associated with symptoms, since that the most of the previous studies considering the survival of restoration as the primary outcome and not pondering the pulp condition. However, the main reason to use the pulp capping material is to protect the pulp tissue. Thus, it should be considered in the evaluation.

Others secondary outcomes will be considered. The choice of outcome measures was based on the efficacy of the treatment, but also in patient-centered outcomes, thinking in about the practice-based evidence where the preference of patient should be englobed in the treatment choice. Analysis of cost will be also performed in order to project the incremental cost of the treatments with higher failure rate for the public health manager.

This is important to highlighted that it will not be possible the blinding of the operators because of the evident differences between the both techniques. Nevertheless, to minimize this situation, the allocation of the treatments will be only performer after the selective caries removal. Furthermore, the patient and examiner can be considered as blinded.

Thus, our study desires to answer if it is possible to restore deep caries lesion of primary teeth only with HVGIC considering the ART philosophy. Since that this hypothesis was sustained, the pediatric dentistry can be used a friendlier technique to deep caries lesion management.

## Additional file

Additional file 1: SPIRIT 2013 Checklist: Recommended items to address in a clinical trial protocol and related documents. (PDF 181 kb)

Additional file 2: CEPECO Collaborative group. (PDF 173 kb)

## Abbreviation

ART: Atraumatic Restorative Treatment
HVGIC: High viscosity glass ionomer cement
