# Supplementary material for: Impact of different restorative treatments for deep caries lesion in primary teeth (CEPECO 1) – study protocol for a noninferiority randomized clinical trial
Source: BMC Oral Health. 2019 Jan 8;19:6. doi: 10.1186/s12903-018-0703-3 (PMC6325681; doi:10.1186/s12903-018-0703-3)

***Center of clinical research in Pediatric Dentistry (CEPECO) collaborative group**

School of Dentistry, Ibirapuera University

Anna Carolina Volpi Mello-Moura (researcher)

Bruna Danielle Pereira (graduation student)

Flávia Maria da Silva Lourenço (graduation student)

Gabriel Koch Moreira da Silva (graduation student)

Gabriela Fernanda Ribeiro Machado (graduation student)

Gabriela Seabra Quennehen da Silva (MSc student)

Gianluca Brezezinski (graduation student)

Jardilene Pereira Conceição (graduation student)

Luana Dias Araújo Santos (graduation student)

Tamara Kerber Tedesco (researcher)

Thais Gimenez (researcher)

School of Dentistry, University of São Paulo, São Paulo, Brazil

Bruna Lorena Pereira Moro (PhD student)

Daniela Prócida Raggio (researcher)

Fausto Medeiros Mendes (researcher)

José Carlos Pettorossi Imparato (researcher)

Laura Regina Antunes Pontes (PhD student)

School of Dentistry, Universidade Cruzeiro do Sul, São Paulo, Brazil

Tatiane Fernandes Novaes (researcher)

School of Dentistry, Universidade Paulista, Campinas, Brazil

Ana Flavia Bissoto Calvo (researcher)

Lucila Basto Camargo (researcher)

Dental Research Center São Leopoldo Mandic, Campinas, Brazil

Rafael Celestino de Souza (researcher)

School of Dentistry, University center of Uninovafapi, Teresina, Brazil

Isabela Floriano (researcher)

School of Dentistry, Federal University of Rio Grande do Sul

Patrícia Daniela Melchiors Angst

School of Dentistry, Cenetista Institute of Higher education of Santo Angelo

Anelise Fernandes Montagner

Dental Health Unit – Williams House, Manchester Science Park, The University of Manchester

Juan Sebastian Lara (researcher)

Organization chart of decision-making process of teeth not included in the trial.

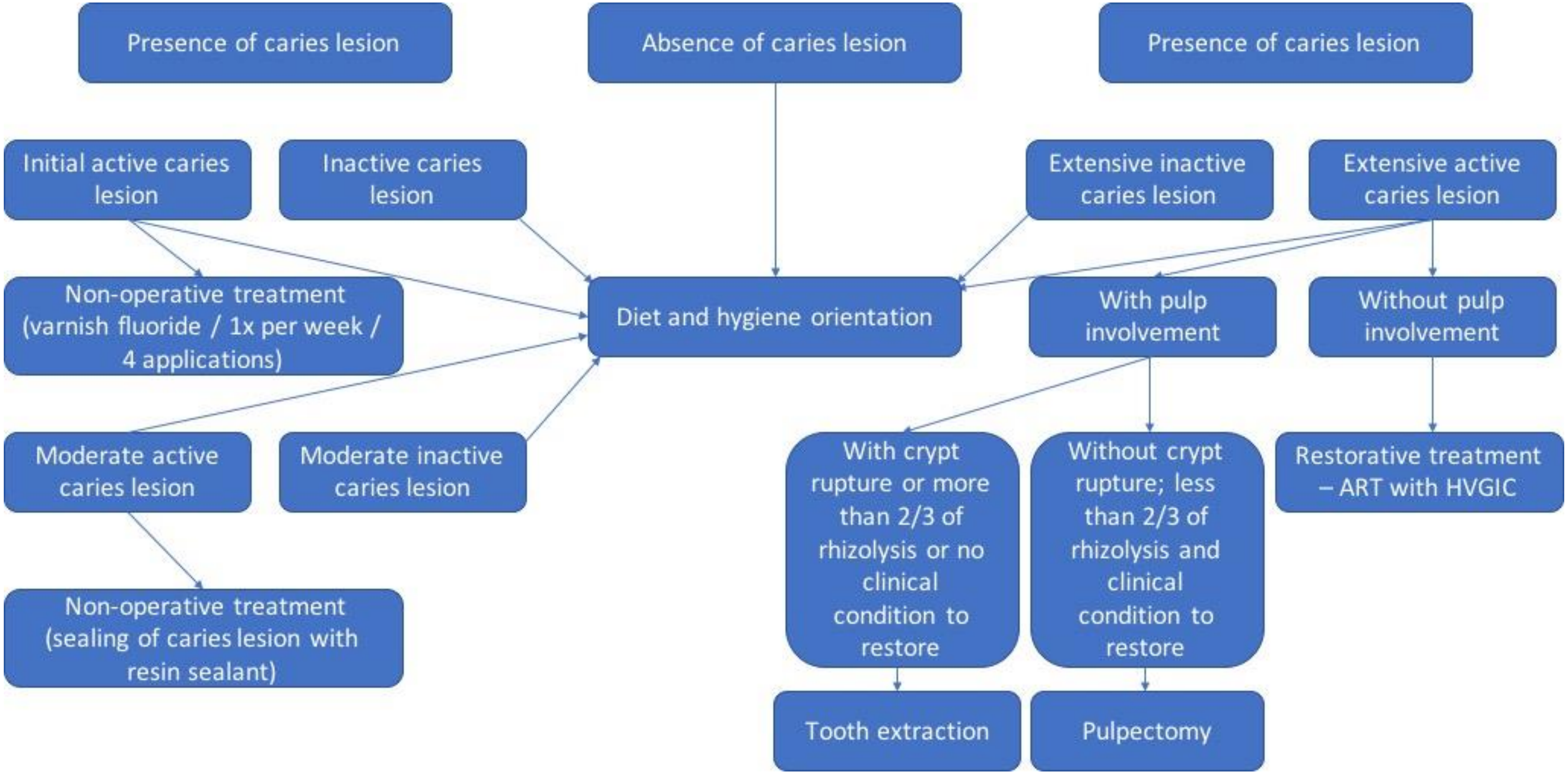

Supplement: Supplementary file 2 — CEPECO Collaborative group. (PDF 173 kb) [file 12903_2018_703_MOESM2_ESM.pdf]
